# Examining the learning curves in robotic cardiac surgery wet lab simulation training

**DOI:** 10.1093/icvts/ivae227

**Published:** 2024-12-30

**Authors:** Gennady V Atroshchenko, Emiliano Navarra, Matthew Valdis, Elena Sandoval, Nasseh Hashemi, Stepan Cerny, Daniel Pereda, Meindert Palmen, Flemming Bjerrum, Niels Henrik Bruun, Martin G Tolsgaard

**Affiliations:** Department of Cardiothoracic Surgery, Aalborg University Hospital, Aalborg, Denmark; ROCnord Robotic Center Aalborg, Aalborg University Hospital, Aalborg, Denmark; Department of Clinical Medicine, Aalborg University, Aalborg, Denmark; Department of Cardiac Surgery, Ospedale San Carlo di Nancy, Rome, Italy; Division of Cardiac Surgery, Department of Surgery, Western University, London, ON, Canada; Department of Cardiovascular Surgery, Hospital Clínic, Barcelona, Spain; Department of Clinical Medicine, Aalborg University, Aalborg, Denmark; Nordsim, Aalborg University Hospital, Aalborg, Denmark; Department of Cardiovascular Surgery, University Hospital Motol, Prague, Czech Republic; Department of Cardiovascular Surgery, Hospital Clínic, Barcelona, Spain; Centro de Investigación Biomédica en Red de Enfermedades Cardiovasculares (CIBERCV), Madrid, Spain; Department of Cardiothoracic Surgery, Leiden University Medical Center, Leiden, the Netherlands; Department of Cardiothoracic Surgery, Amsterdam University Medical Center, Amsterdam, the Netherlands; Gastrounit, Surgical Section, Copenhagen University Hospital—Amager and Hvidovre, Hvidovre, Denmark; Copenhagen Academy for Medical Education and Simulation (CAMES), Center for HR & Education, Copenhagen, Denmark; Department of Clinical Medicine, Faculty of Health and Medical Sciences, University of Copenhagen, Copenhagen, Denmark; Unit of Clinical Biostatistics, Aalborg University Hospital, Aalborg, Denmark; Copenhagen Academy for Medical Education and Simulation (CAMES), Center for HR & Education, Copenhagen, Denmark; Department of Clinical Medicine, Faculty of Health and Medical Sciences, University of Copenhagen, Copenhagen, Denmark; Department of Obstetrics, Copenhagen University Hospital Rigshospitalet, Copenhagen, Denmark

**Keywords:** robotic cardiac surgery, wet lab simulation, learning curves, surgical training

## Abstract

**BACKGROUND:**

Simulation-based training has gained distinction in cardiothoracic surgery as robotic-assisted cardiac procedures evolve. Despite the increasing use of wet lab simulators, the effectiveness of these training methods and skill acquisition rates remain poorly understood.

**OBJECTIVES:**

This study aimed to compare learning curves and assess the robotic cardiac surgical skill acquisition rate for cardiac and noncardiac surgeons who had no robotic experience in a wet lab simulation setting.

**METHODS:**

In this prospective cohort study, participants practiced 3 robotic tasks in a porcine model: left atriotomy closure, internal thoracic artery harvesting and mitral annular suturing. Participants were novice robotic cardiac and noncardiac surgeons alongside experienced robotic cardiac surgeons who established performance benchmarks. Performance was evaluated using the time-based score and modified global evaluative assessment of robotic skills (mGEARS).

**RESULTS:**

The participants were 15 novice surgeons (7 cardiac; 8 noncardiac) and 4 experienced robotic surgeons. Most novices reached mastery in 52 (±22) min for atrial closure, 32 (±18) for internal thoracic artery harvesting and 34 (±12) for mitral stitches, with no significant differences between the cardiac and noncardiac surgeons. However, for mGEARS, noncardiac novices faced more challenges in internal thoracic artery harvesting. The Thurstone learning curve model indicated no significant difference in the learning rates between the groups.

**CONCLUSIONS:**

Wet lab simulation facilitates the rapid acquisition of robotic cardiac surgical skills to expert levels, irrespective of surgeons’ experience in open cardiac surgery. These findings support the use of wet lab simulators for standardized, competency-based training in robotic cardiac surgery.

## Graphical Abstract

**Figure:**
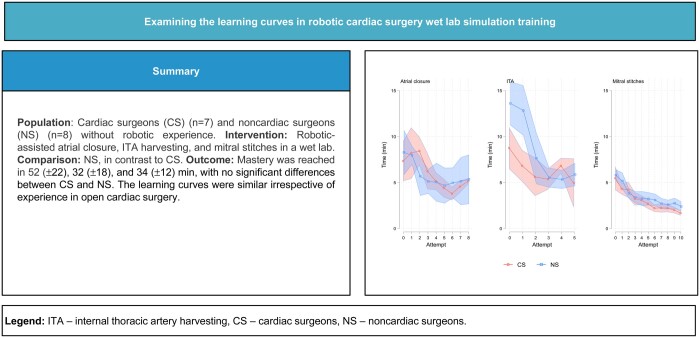

## INTRODUCTION

In recent years, focus on simulation-based training in cardiothoracic surgery has increased significantly [1, 2]. As surgery advances by adopting less-invasive technologies and becoming more technically challenging, along with concerns about improved patient safety, gaining competency in the preclinical setting has become of major importance [3, 4]. Robotic-assisted cardiac surgery is a rapidly evolving field of minimally invasive surgery initially limited to mitral valve [5] and coronary artery bypass grafting [6]; recently, it expanded to include aortic valve replacement [7], atrial fibrillation treatment[8], etc. Although simulation is an essential part of modern robotic surgical training [9, 10], its effectiveness is not well investigated within cardiac surgery [11].

Learning curves are increasingly used in the assessment of competency and design of training programmes [12, 13]. In surgery, methods currently used to analyse learning curves are mainly descriptive and must be rigorous, with quantifiable parameters, to allow future surgeons to benefit from established performance standards [14]. Despite available learning curves data in robotic cardiac procedures in real-world environments [15–17], little, if anything, is known about the robotic cardiac skill acquisition rate in a wet lab simulation setting [18]. Moreover, a common perception is that surgeons must be expert in nonrobotic cardiac operations before performing robotic-assisted surgery [19]. However, expertise in video-assisted thoracoscopic surgery does not shorten the learning curve of robotic-assisted thoracic surgery [20], and transfer from laparoscopic to robot-assisted surgery is not evident [21]. As robotic-assisted surgery is expected to become the first-line modality for many future surgeons [21], the role of nonrobotic experience must be clarified. Thus, this study aimed to examine learning curves and assess the robotic cardiac surgical skill acquisition rate for cardiac and noncardiac surgeons without robotic experience, aiming for mastery in a wet lab simulation setting. To the best of our knowledge, this study is the 1st to analyse the learning curves of robotic cardiac surgical skills in wet labs for participants with different surgical backgrounds, comprising the largest number of participants to date.

## MATERIALS AND METHODS

### Study design

This was a prospective study gathering data from a 4-week simulation training at the Biomedical Research Laboratory, Aalborg University, Denmark. The study was registered at ClinicalTrials.gov (NCT05043064), and an exemption for approval before trial enrolment was obtained from the Regional Scientific Ethical Committee of the North Denmark Region (Reference no. 2020-000992-55). All participants signed an informed consent form before participating in the study.

### Participants

Surgeons and surgical trainees with different specialities were invited to participate in the study and allocated into 2 groups: novice robotic cardiac and robotic noncardiac surgeons. A group of expert robotic cardiac surgeons was enrolled to establish the performance standards. Novice robotic surgeons who had completed or had ongoing surgical speciality residency and <5 h of experience in any robotic system were included. The novice surgeons were recruited by e-mail invitation sent to different surgical departments in Denmark between August and September 2021 on a first-come, first-served basis. Expert robotic cardiac surgeons who had performed a minimum of 50 robotic cardiac operations as console surgeons were also included. Participants were recruited from different European sites by personal e-mail inquiry between January and April 2021.

### Intervention

**Figure 1:**
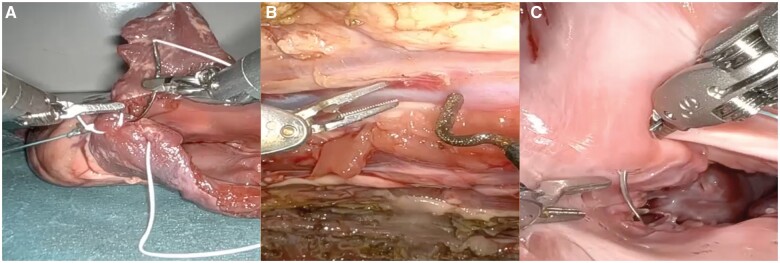
Wet lab simulation tasks. (**A**) Robotic-assisted closure of the left atrium. (**B**) Robotic-assisted harvesting of the internal thoracic artery. (**C**) Robotic-assisted placement of the mitral annular stitches.

The study was conducted in a simulated environment using the da Vinci Xi Surgical System (Intuitive Surgical, CA, USA). Three wet lab tasks were chosen for the study (Fig. 1):

1. Robotic-assisted closure of an 8-cm-long left atriotomy in an isolated porcine heart.
2. Robotic-assisted harvesting of a 10-cm segment of the internal thoracic artery (ITA) off the prepared porcine anterior chest wall.
3. Robotic-assisted placement of 5 annular sutures in the mitral annulus of an isolated porcine heart.

The model and tasks were described in detail with a video in a previous study [22]. Two scores were used to assess participants’ performance: a time-based score (TBS) and a modified global evaluative assessment of robotic skills [23] (mGEARS) score. Both scores and their validity evidence for the assessment of robotic-assisted cardiac surgery skills in wet lab settings were previously described in greater detail [22]. Briefly, TBS was calculated as follows: TBS = maximal total training time per task − actual completion time − errors. The idea behind the TBS was not solely to concentrate on the actual time spent on the task but also to be able to adjust for the defined set of errors, such as gross tissue damage or torn sutures. TBS was calculated in seconds, where 0 was the lowest score and a higher number indicated a faster time to perform the task. The mGEARS score was used to evaluate the overall robotic proficiency by 5 domains: depth perception, bimanual dexterity, efficiency, force sensitivity, and robotic control. The score ranged from 5 to 25, where 5 and 25 indicated the least and most proficient performance, respectively. The mGEARS did not account for time.

The expert robotic cardiac surgeons were asked to perform each task 5 times. The mean of their last 2 attempts was used as the mastery learning level for each task by TBS and mGEARS scores. The novice surgeons were asked to complete the 3 tasks once, which was used as their baseline performance. Then, the novice surgeons continued to do the tasks until the mastery based on the TBS of the experienced surgeons was achieved. To ensure the successful task completion, each participant was required to pass the exercise 2 times consecutively. Training sessions were 1.5 h with an interval of 2 days–3 weeks between sessions [24]. During the study, the novice surgeons were not allowed to practice on any robotic simulators or participate in robotic-assisted surgery. All tasks were videorecorded. The TBS was rated on site during task performances. The mGEARS score was obtained using a blinded video rating by 2 robotic cardiac surgeons not involved in the task performance.

### Outcomes

The primary outcome was the total training time for the novice surgeons to reach the mastery learning level for each task based on the TBS.

### Statistics

Descriptive categorical data are reported as counts and percentages and descriptive continuous data as median with lower and upper quartiles. Categorical data were compared using chi-square tests and continuous data using the Kruskal–Wallis test. Baseline performances, time spent and number of attempts between novice surgeons are expressed as means and standard deviations. Comparisons were done by *t*-tests. The mean differences with 95% confidence intervals and *P*-values for the lack of difference were reported. The individual learning curves were graphed, and a lowess smoothing curve was added. A mixed-effect maximum likelihood nonlinear regression with a random intercept by each participant was used to analyse the Thurstone learning curves [25]. The significance level was 5%. STATA version 18 was used for data management and analyses.

## RESULTS

Seven novice robotic cardiac surgeons from 3 different Danish departments of cardiothoracic surgery were recruited. Eight novice robotic noncardiac surgeons from the Danish departments of abdominal surgery (*n* = 4), urology (*n* = 1), gynaecology (*n* = 2) and otolaryngology (*n* = 1) were recruited. The cardiac and noncardiac surgeons were comparable by age, sex, number of specialists and years of experience in the speciality (Table 1). Four experienced robotic cardiac surgeons were recruited to establish the mastery learning level.

**Table 1:** Descriptive data on the study participants

Characteristic	Novice cardiac surgeons	Novice noncardiac surgeons	Total	*P*-value
*N*	7	8	15	
Age (years), median (IQR)	55 (43; 63)	42 (36; 48)	47 (37; 56)	0.2
Sex (female), *n*	2	3	5	0.71
Specialist (yes), *n*	6	6	12	0.6
Work experience (years), median (IQR)	14 (3; 21)	4.5 (2; 9)	5 (2; 17)	0.3

IQR: interquartile range.

The mastery learning level by TBS was calculated as follows: TBS = maximal total training time per task − expert completion mean time − errors. The results were as follows:

Atrial closure = 769 − 190 − errors = 524 s

ITA harvesting = 1222 − 347 − errors = 862 s

Mitral stitches = 566 − 79 − errors = 450 s

The mastery learning level by mGEARS was indicated by scores of 19 for atrial closure, 18 for ITA harvesting and 19 for the mitral stitch task.

No significant difference was found in the mean TBS at baseline between novice robotic cardiac and noncardiac novices for all the tasks (Table 2). However, the novice robotic cardiac surgeons scored higher on mGEARS for the ITA dissection task at baseline than the novice robotic noncardiac surgeons (*P* = 0.01).

**Table 2:** Comparison of the performance of novice cardiac and noncardiac surgeons at baseline

Score	Task	Novice cardiac surgeons, mean±SD	Novice noncardiac surgeons, mean±SD	Effect, mean difference (95% CI)	*P*-value
TBS (s)	Atrial closure	127 ± 171	143 ± 181	− 16 (−200; 168)	0.86
	ITA	589 ± 303	322 ± 250	267 (−25; 560)	0.07
	Mitral stitches	161 ± 128	119 ± 91	42 (−74; 160)	0.46
mGEARS	Atrial closure	13 ± 2	11 ± 3	2 (−1; 4)	0.16
	ITA	13 ± 2	10 ± 0	2 (1; 4)	0.01
	Mitral stitches	11 ± 3	11 ± 3	0 (−2; 3)	0.74

CI: confidence interval; mGEARS: modified global evaluative assessment of robotic skills score; SD: standard deviation.

Overall, 435 task repetitions were performed during the study. Out of 15 novice participants, 13 (7 cardiac and 6 noncardiac) achieved the mastery learning level in all 3 tasks on the TBS. Of the 2 remaining novice robotic noncardiac surgeons, 1 achieved mastery in both mitral stitching and atrial closure, and 1 achieved proficiency only in mitral suture placement. The reason for the former was the inability to reach mastery within scheduled training sessions and for the latter was the participant’s inability to attend the scheduled training sessions because of logistical issues.

**Figure 2:**
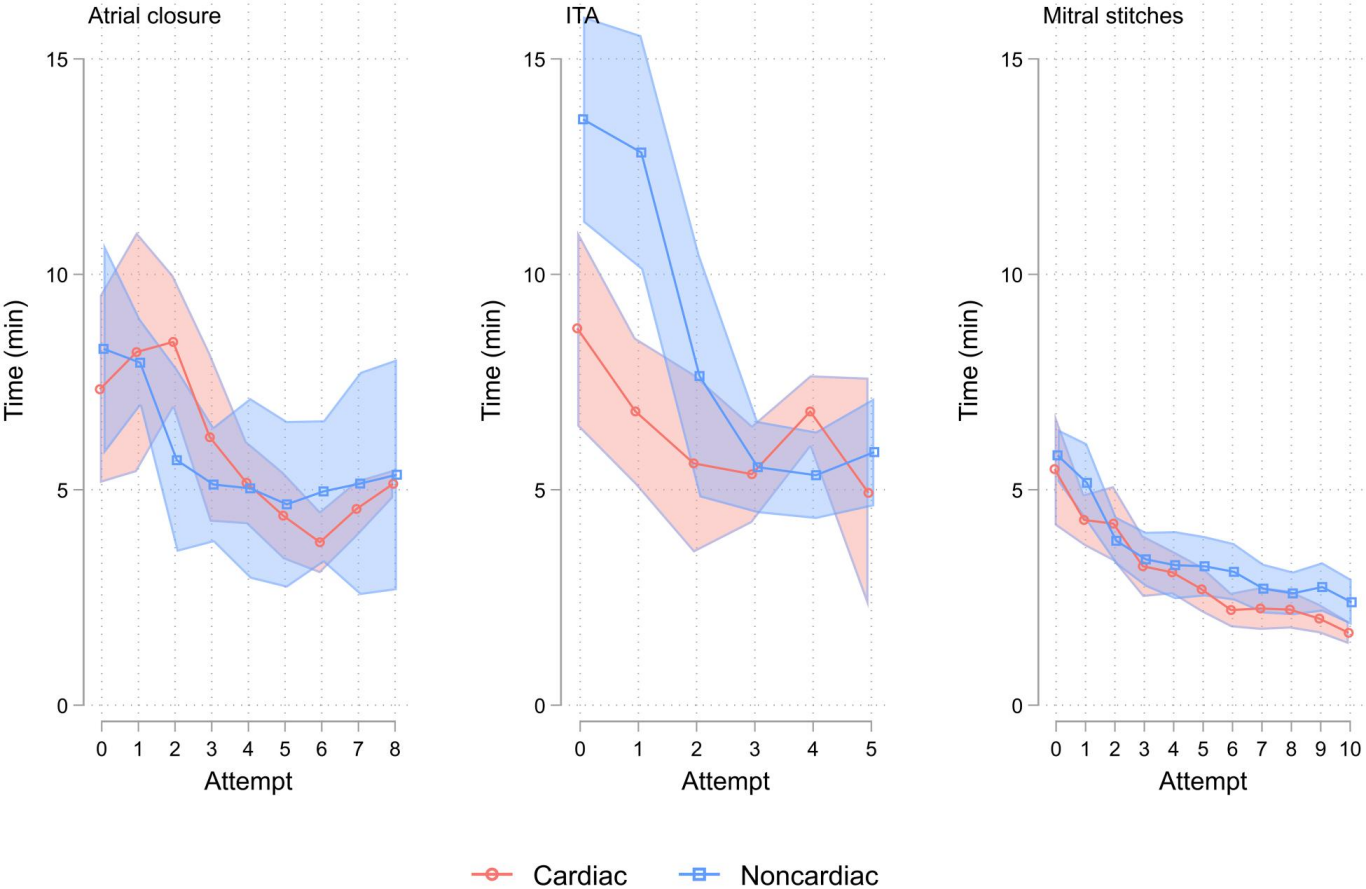
Time spent on the task during consecutive attempts in both novice groups. Groups’ median time with 95% confidence interval. Cardiac: robotic cardiac novice group; ITA: internal thoracic artery; Noncardiac: robotic noncardiac novice group.

**Figure 3:**
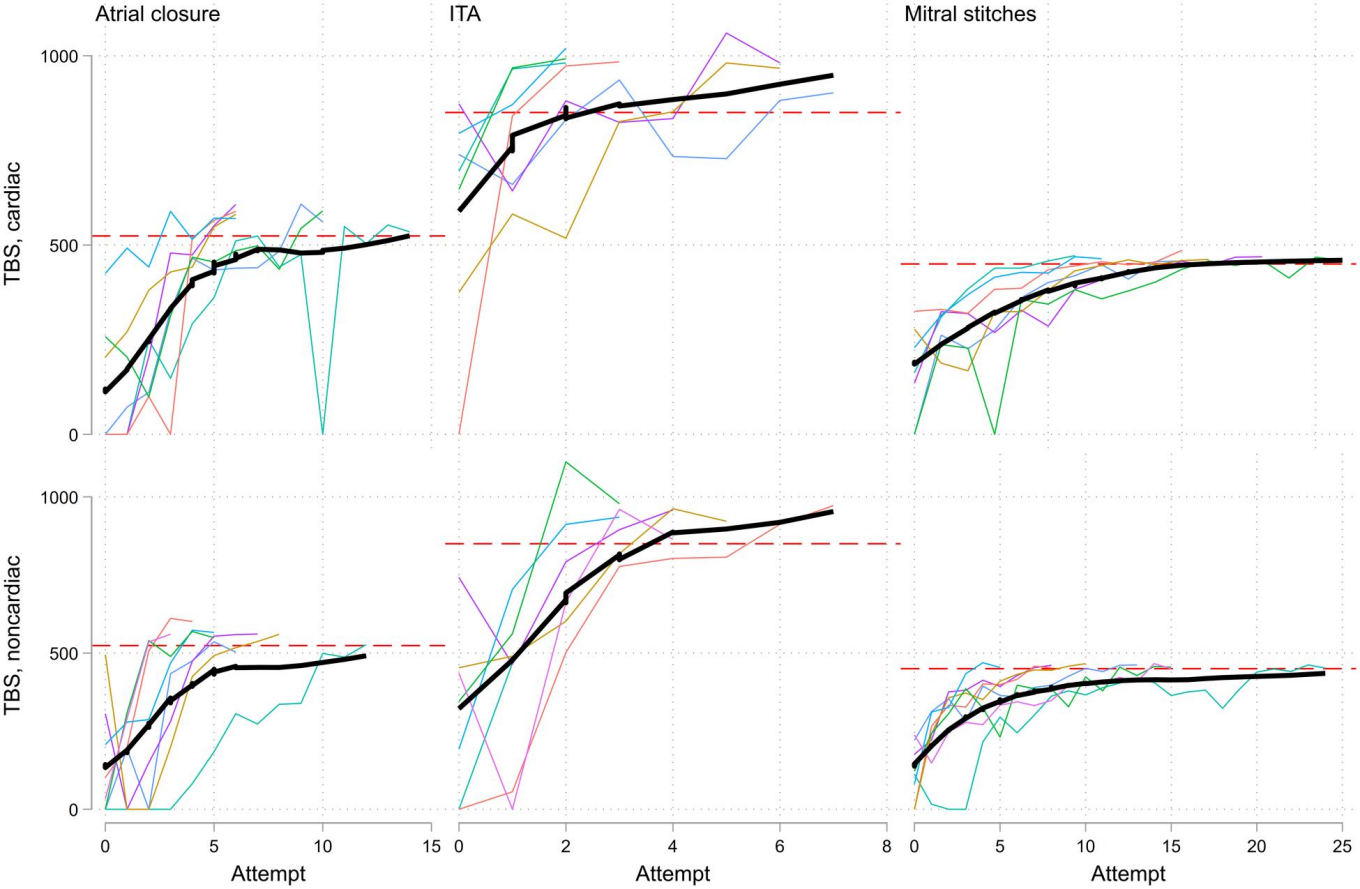
Individual learning curves based on the TBS. The red dashed line refers to the mastery learning level. The thick black curve is the lowess estimated curve through the points. ITA: internal thoracic artery; TBS: time-based score.

The average time and number of attempts to reach the mastery learning level on TBS are demonstrated in Table 3. Neither of them differed significantly between the cardiac and noncardiac surgeons for all 3 tasks. The median time spent on the task declined sharply over the first 3 repetitions, which then levelled off for atrial closure and ITA dissection and continued to decline smoothly for mitral stitching (Fig. 2). Individual performance curves demonstrated a similar pattern in achieving mastery on TBS in both groups (Fig. 3).

**Table 3:** Time and number of attempts to reach mastery in the cardiac and noncardiac groups

	Task	Novice cardiac surgeons, mean±SD	Novice noncardiac surgeons, mean±SD	Effect, mean difference (95% CI)	*P*-value
Time (min)	Atrial closure	52 ± 22	48 ± 30	4 (−24; 31)	0.79
	ITA	32 ± 18	40 ± 18	−8 (−27; 10)	0.35
	Mitral stitches	34 ± 12	45 ± 23	−11 (−30; 8)	0.25
Attempts (*n*)	Atrial closure	8 ± 3	6 ± 3	2 (−1; 5)	0.2
	ITA	4 ± 2	3 ± 2	1 (−2; 3)	0.59
	Mitral stitches	10 ± 3	12 ± 6	−2 (−7; 3)	0.47

CI: confidence interval; SD: standard deviation.

Five cardiac (out of 7) and 2 noncardiac novice surgeons (out of 7) reached the determined mastery learning level based on the mGEARS score 2 consecutive times for the atrial closure task (*P* = 0.11). Significantly fewer (*P* = 0.03) novice noncardiac surgeons (3 out of 6) achieved mastery by the mGEARS score for ITA dissection compared with novice cardiac surgeons (7 out of 7). All novice cardiac surgeons (7 out of 7) and 7 out of 8 novice noncardiac surgeons mastered the mitral stitch task based on the mGEARS score (*P* = 0.33).

**Figure 4:**
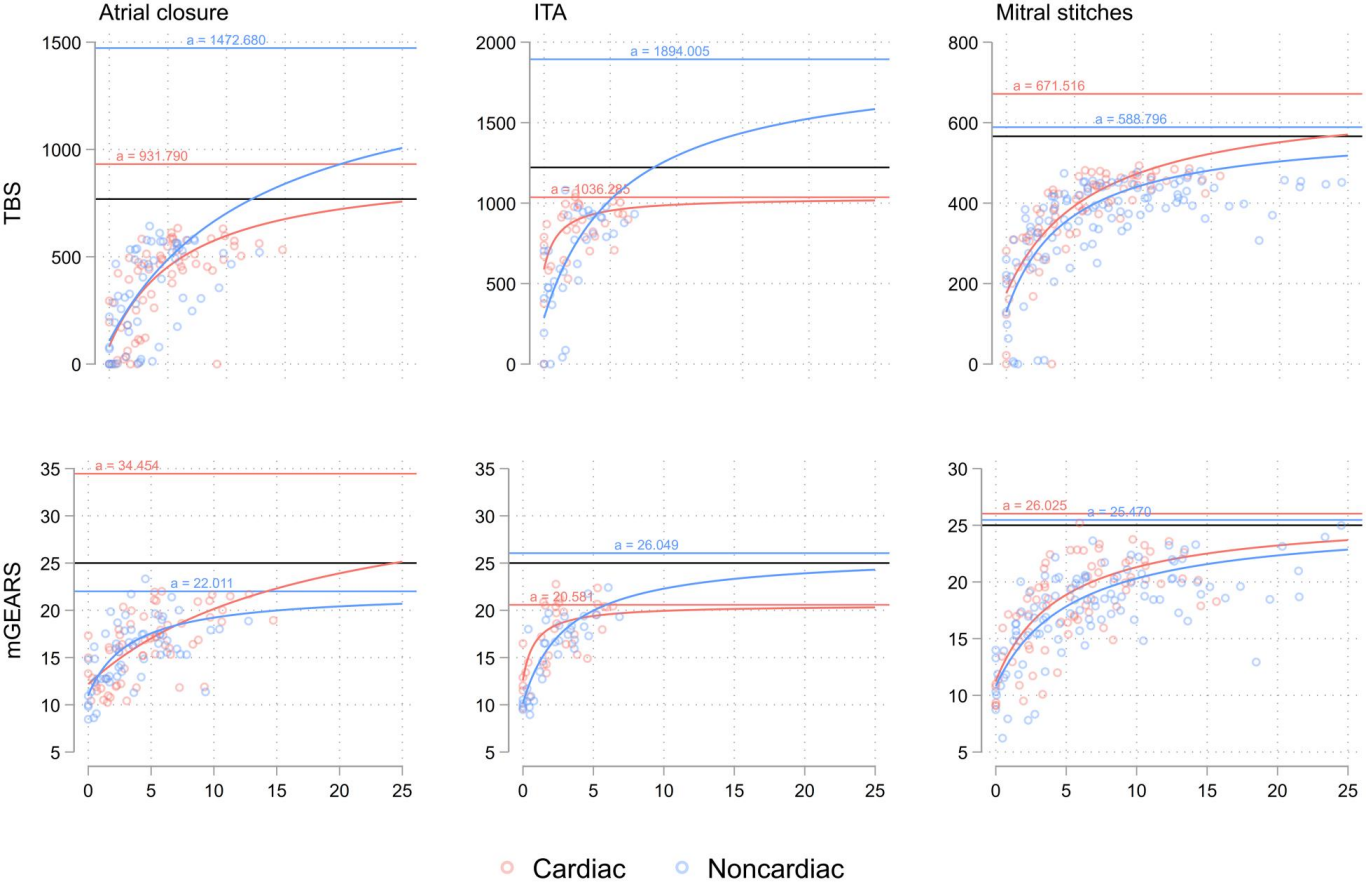
Group-level Thurstone learning curves for the TBS and mGEARS scores. The black solid line is the maximum attainable score: 769 for atrial closure, 1222 for ITA and 566 for mitral stitches on TBS and 25 for the mGEARS score. ITA: internal thoracic artery; mGEARS: modified global evaluative assessment of robotic skills score.

Using the Thurstone learning curve model [25] for the group level comparison of both robotic novice groups, the initial level of performance (y-intercept), learning rate (slope) and maximum possible learning (asymptote) were estimated. No significant difference was found in the estimates between the 2 groups, except for the y-intercept for the ITA harvesting task, where novice robotic cardiac surgeons performed better based on both TBS and mGEARS score (Table 4). The hypothetical probability of reaching the maximum achievable score is demonstrated in Fig. 4. As per the model, the maximum score of 25 on mGEARS at a group level could not be reached even with continuous training by novice noncardiac surgeons for the atrial closure task and by the novice cardiac surgeons for the ITA dissection task because their asymptotes were <25. Similarly, for TBS, the maximum attainable score of 1222 s in the Thurstone modelling could not be reached by novice cardiac surgeons because their estimated maximum possible learning was 1036 s.

**Table 4:** Comparison of the group-level performance of novice cardiac and noncardiac surgeons using the Thurstone learning model

Score	Task	Estimate	Novice cardiac surgeons, mean (96% CI)	Novice noncardiac surgeons, mean (96% CI)	*P*-value
TBS (s)	Atrial closure	y-intercept	83 (−31; 197)	107 (2; 212)	0.76
		Slope	6 (0; 13)	13 (−4; 30)	0.5
		asymptote	932 (540; 1323)	1473 (292; 2653)	0.39
	ITA	y-intercept	588 (443; 734)	286 (153; 418)	<0.001
		slope	1 (−1; 3)	6 (−3; 15)	0.3
		asymptote	1036 (766; 1306)	1894 (496; 3292)	0.24
	Mitral stitches	y-intercept	176 (125; 227)	129 (80; 177)	0.19
		slope	6 (2; 10)	5 (3; 7)	0.42
		asymptote	671 (536; 807)	589 (515; 662)	0.29
mGEARS	Atrial closure	y-intercept	12 (11; 14)	11 (9; 13)	0.34
		slope	18 (−13; 49)	3 (−1; 7)	0.37
		asymptote	34 (9; 60)	22 (17; 27)	0.35
	ITA	y-intercept	13 (11; 14)	10 (9; 12)	0.03
		slope	1 (−1; 2)	3 (−1; 6)	0.2
		asymptote	21 (18; 23)	26 (18; 34)	0.18
	Mitral stitches	y-intercept	11 (9; 13)	11 (9; 13)	0.74
		slope	5 (1; 8)	5 (2; 9)	0.76
		asymptote	26 (22; 30)	25 (22; 29)	0.83

Asymptote: maximum possible learning; CI: confidence interval; mGEARS: modified global evaluative assessment of robotic skills score; TBS: time-based score; y-intercept: initial level of performance; slope: rate of learning.

## DISCUSSION

This study on the robotic wet lab simulation, the largest of its kind, established the mastery learning level using TBS and mGEARS scores across 3 robotic-assisted tasks in a porcine model. The robotic skill acquisition rate in 2 groups of surgeons without robotic experience, namely, cardiac surgeons and surgeons form other specialties, were explored. Our findings indicate that wet lab simulation enables the rapid acquisition of robotic cardiac skills to an expert level for both groups. Qualitative and quantitative analyses of the learning curves were performed, and no significant differences were observed between the groups. This suggests that robotic surgical skills are procedure-specific and independent of open cardiac surgical experience [21]. These findings highlight the value of using mastery learning in robotic cardiac surgery training programmes. Such training can also help in identifying learners who may require additional support and allow for the adjustment of training regimens accordingly.

The classic stepwise educational approach in robotic cardiac surgery involves increasing entrustment with increasing levels of surgical skills [11, 26]. However, this approach is not efficient because it mainly relies on clinical training with little use of simulation-based training as the initial part of the entrustment process. Although nearly all robotic cardiac training programmes employ simulation training [9, 10] with the assumption that the time spent on simulation modifies the clinical learning curve [27], neither the content nor the standard level of competency in such training are defined. Currently, 3 modalities are used in robotic surgical training: dry labs, virtual reality simulation and wet labs in cadaveric or animal models [28]. Skill acquisition rates using these modalities in cardiac surgery are very scarce; however, procedure-specific wet lab robotic simulation appears to be associated with the fastest skill acquisition [29]. Even though wet lab simulation has some well-documented limitations, such as high costs, logistical challenges and restricted access, it remains to be an essential part of robotic cardiac training [30], and experts recommend practicing in porcine model before initial cases [31]. Previously, only 1 study examined proficiency levels in robotic cardiac surgery simulation-based training [29]. In contrast, the present study not only included a larger cohort of expert surgeons but also encompassed a broader range of tasks. The use of validated scores [22] enhances the reliability of the mastery learning levels defined in our approach and supports the scalability of our training setup for robotic-assisted coronary revascularization and mitral valve surgery.

Notably, the novice cardiac and noncardiac surgeons achieved mastery within 32–52 min over ∼12 repetitions for all tasks. This efficiency aligns with the typical duration of short-course robotic cardiac wet lab training sessions provided by professional societies [32]. Interestingly, although the difference was not significant, it appeared that noncardiac surgeons reached mastery faster in tasks such as atrial closure, suggesting that some skills may be transferable across different surgical disciplines. However, differences emerged in the mGEARS score; in particular, the ITA harvesting task appeared to be more challenging for novice noncardiac surgeons. This pattern highlights the task-dependent nature of learning curves and the necessity for multiple iterations to achieve mastery in more complex tasks.

Surgeon expertise in performing routine and high-risk cardiac procedures before proceeding to robotic-assisted surgery has been deemed essential [19, 33]. In this study, despite significant experience with all 3 tasks as a part of the traditional open-chest surgery routine with a median work experience of 14 years, cardiac surgeons did not show any significant difference in robotic performance either at the baseline or in the pace of learning compared with noncardiac surgeons. Our use of the Thurstone learning curve model for a quantitative group-level analysis confirmed a consistent rate of learning across both groups for all tasks, emphasizing that robotic skills acquisition is not dependent on surgical experience from open chest surgery. This finding supports the integration of robotic training early in cardiothoracic surgery residency, potentially shortening the learning curve in clinical settings. As robotic simulators evolve, the ‘open’ experience gap may be substantially reduced [17].

Implementing scoring systems in robotic skills training is urgently needed to demonstrate the learning process and facilitate proficiency-based progression [34]. The simulation model and the scales used in this study could help in establishing a single standardized evaluation of robotic cardiac skills across different institutions. Thus, tangible assessment parameters are necessary for a console surgeon, and centres deploying wet lab simulation are suggested to incorporate our model. Training should continue until the pass/fail level [22] at either TBS or mGEARS is achieved. Ideally, a trainee should practice until attaining mastery learning level. However, expanding this type of training across institutions will likely encounter limitations for multiple reasons, such as infrastructures, costs, access to animal models and availability of the robotic platform for training (probably the most limiting one). Perhaps, a more realistic way would be to concentrate training to a few centres with resources and infrastructure and offer the training to the entire community of residents, fellows and surgeons. This would make it more sustainable and ensure homogeneity in terms of methodology and results.

### Strengths and limitations

The major strength of this study lies in the use of assessment tools such as TBS and mGEARS supported by validity evidence, enhancing the reliability of our learning curve analysis. However, the small participant size raises the possibility of a Type II error, proposing cautious interpretation of our findings. Nevertheless, the consistency of our results across multiple analyses strengthens the hypothesis that robotic cardiac surgery skills are specific to the procedure rather than influenced by surgical experience.

## CONCLUSIONS

Wet lab simulation provides fast acquisition of certain robotic cardiac surgical skills in surgeons with no robotic experience to the expert level. The learning curves of robotic cardiac skills do not differ between cardiac and noncardiac surgeons. Robotic cardiac surgical skills appear to be procedure-specific, and the performance curve for these skills does not reflect cardiac surgical experience.

## Data Availability

The data underlying this article will be shared on reasonable request to the corresponding author.

## ABBREVIATIONS

ITA: Internal thoracic artery
mGEARS: Modified global evaluative assessment of robotic skills
TBS: Time-based score
